# SVD-Based Technique for Interference Cancellation and Noise Reduction in NMR Measurement of Time-Dependent Magnetic Fields

**DOI:** 10.3390/s16030323

**Published:** 2016-03-04

**Authors:** Wenjun Chen, Hong Ma, De Yu, Hua Zhang

**Affiliations:** 1 School of Physics, Huazhong University of Science and Technology, Wuhan 430074, China; chenwj1025@hust.edu.cn (W.C.); yude@hust.edu.cn (D.Y.); 2 School of Electronics Information and Communication, Huazhong University of Science and Technology, Wuhan 430074, China; mahong@hust.edu.cn

**Keywords:** singular value decomposition, magnetic field measurement, time-dependent magnetic fields, interference cancellation, noise reduction

## Abstract

A nuclear magnetic resonance (NMR) experiment for measurement of time-dependent magnetic fields was introduced. To improve the signal-to-interference-plus-noise ratio (SINR) of NMR data, a new method for interference cancellation and noise reduction (ICNR) based on singular value decomposition (SVD) was proposed. The singular values corresponding to the radio frequency interference (RFI) signal were identified in terms of the correlation between the FID data and the reference data, and then the RFI and noise were suppressed by setting the corresponding singular values to zero. The validity of the algorithm was verified by processing the measured NMR data. The results indicated that, this method has a significantly suppression of RFI and random noise, and can well preserve the FID signal. At present, the major limitation of the proposed SVD-based ICNR technique is that the threshold value for interference cancellation needs to be manually selected. Finally, the inversion waveform of the applied alternating magnetic field was given by fitting the processed experimental data.

## 1. Introduction

Nuclear magnetic resonance (NMR) is usually performed in a stable magnetic field. However, in many cases, we need to pursue NMR in a time-dependent magnetic field; for instance, the field induced normal state in high *T*_c_ superconductors lies above 100 T, but such fields can only be produced temporarily by a pulsed magnet [1,2,3]. To study the NMR experimental technique in time-dependent magnetic fields as well as the related data processing method, a ^1^H-NMR experiment was conducted in an alternating magnetic field in this work. However, affected by some home-made devices in our setup and the experimental environment, there is non-negligible radio frequency interference (RFI) in the measured NMR data. As a result, the signal to interference plus noise ratio (SINR), which defined as the power of the free induction decay (FID) signal divided by the sum of the RFI power and the power of background noise, is rather low; in certain data sets, the spectral peak amplitude of RFI is even higher than that of FID signal, which will lead to confusion between the interference peaks and the FID signal spectral peak, and cause a deviation in the magnetic field fitting. Therefore, we have to employ an appropriate interference cancellation algorithm to identify and remove the RFI.

There is no “universal” interference cancellation algorithm. For a specific RFI, various interference cancellation algorithms have different effects. If the RFI is a narrow-band and stationary signal, a simple notch filtering method can adequately remove the interference [4]. However, the frequency-domain notch filtering algorithm requires a priori information or high accurate frequency estimation of RFI. Furthermore, this method does not suffice in more complex RFI environments, for example, where the frequency of RFI changes over time.

A more common approach of performing interference cancellation is the adaptive cancelling scheme. The representative adaptive algorithms (e.g., LMS, NLMS, and RLS) have been applied in the time domain [5,6] as well as various transform domains [7,8,9]. In most cases, however, these adaptive algorithms require an iterative process to obtain the optimal filtering weight coefficient, resulting in a high computational complexity and a large amount of calculation time.

In addition, some interference cancellation algorithms based on singular value decomposition (SVD) method are reported [10,11]. These algorithms are mainly applied in processing synthetic aperture radar (SAR) data. In this case, after SVD, the SAR signal has small and similar order of magnitude singular values as the characteristic of white noise, whereas the RFI merely corresponds to several dominant singular values. The RFI can be estimated using these largest singular values, and subtracted from the measured data. The SVD-based algorithm described in these articles can effectively suppress the time-variant RFI. However, they are only applicable to remove the narrow band interference (NBI) from a wideband signal and suitable for the situation where the interference power is much higher than the interested signal.

In this article, the NMR experimental setup and scheme in a sinusoidal alternating magnetic field are introduced first. Then, in order to rapidly suppress the RFI, whose power level is comparable with that of the FID signal, a new interference cancellation and noise reduction (ICNR) algorithm based on singular value decomposition is proposed. After the ICNR processing, the SINR of NMR data was improved significantly. At the end, the waveform of the magnetic field was given by data fitting.

## 2. NMR in Time-Dependent Magnetic Fields

In this work, we employ a routine pulsed NMR technique to detect NMR signal in a sinusoidal alternating magnetic field. Our experimental setup comprises a magnet, an NMR probe and associated electronics devices.

The alternating magnetic field *B_z_* is generated by a hybrid magnet, which consists of a permanent magnet and an electromagnet. The field strength of the permanent magnet *B_z_*_0_, measured by a gauss meter based on a Hall effect sensor, is about 0.579 T. The gyromagnetic ratio γ of the ^1^H nucleus is 2.6752 × 10^8^ rad·s^−1^·T^−1^, the corresponding Larmor frequency *f*_0_ is about 24.652 MHz. The electromagnet produces an additional alternating magnetic field ${\tilde{B}}_{z}$. The operating voltage of the electromagnet can be adjusted in the range of 0–250 V. When operating at the maximum voltage 250 V, the amplitude of the additional alternating magnetic field *B_zp_* is about 0.012 T. Thus, the bandwidth Δf($=\gamma \cdot 2B_{zp}/2\pi$) of the RF excitation signal, NMR probe and receiver should be at least 1.02 MHz. Assuming $\omega_{z}$ and $\varphi_{z0}$ are the actual angular frequency and initial phase angle of the alternating magnetic field, the strength of the alternating magnetic field at time *t* is:

(1) $$B_{z}(t)=B_{z0}+B_{zp}\sin(\omega_{z}t+\varphi_{z0})$$

The basic principle of NMR measurement is the measurement of Larmor precession frequency. Nuclear magnetic resonance will occur when the frequency *f*_1_ of the RF excitation field is exactly equal to the Larmor frequency *f*_0_ [12] (p. 11). By measuring the spectral peak frequency of each FID signal, we can calculate the corresponding magnetic field strength $B_{z}(t)$ at the moment of pulsed excitation. Because of the rate of change of the alternating magnetic field is slow (nearly 50 Hz), the frequency and phase of FID signal modulated by the change of the magnetic field have be ignored. Then, all parameters of the alternating magnetic field can be estimated by fitting the measured data points according to Equation (1) using a least squares method. The accuracy of the magnetic field measurement is mainly determined by the precision of the measurement frequency of each FID signal.

The NMR probe used in our experiment is a home-made single coil probe. A solenoid coil of 10 turns is formed by wrapping a 30-gauge enameled copper wire upon a glass tube (5-mm outer diameter, 8-mm length). The coil pitch is adjusted to be approximately equal to the wire diameter and then fixed with epoxy resin. The resistance and inductance of this solenoid coil is about 0.95 Ohm and 0.75 µH, respectively. In order to transmit RF power to the NMR probe efficiently, the probe impedance has been matched to the characteristic impedance of the coaxial cable ($Z_{0}=50$ Ohm). There are several types of impedance matching network [13,14]. A routine L-type coupling network is used for this purpose due to its simple structure and high efficiency. The quality factor Q, with the sample in place, is about 35, and the bandwidth of the probe is nearly 2 MHz.

**Figure 1:**
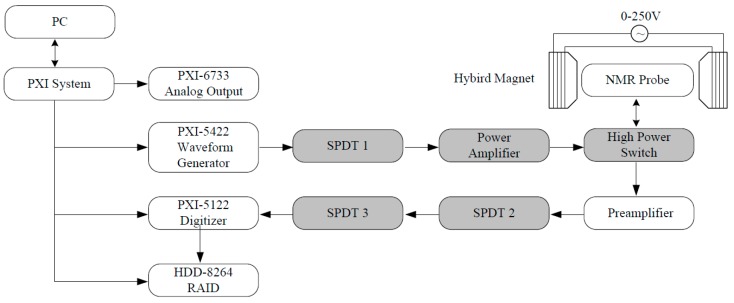
Schematic diagram of the NMR system, all gray parts are controlled by PXI-6773.

The sketch of our NMR system is shown in Figure 1. The experimental setup is built around a National Instruments (NI) PXI system [15]. The PXI system is controlled by a high-bandwidth, 2.3 GHz Dual-Core Embedded Controller (NI PXIe-8130, National Instruments, Austin, TX, USA). High speed analog output (NI PXI-6733, National Instruments, Austin, TX, USA) is used to control various single pole double throw (SPDT) switches (2ASWA-2-50DR+, Mini-circuits, New York, NY, USA), whose maximum RF input power is only 24 dBm, a home-made high power SPDT switch (the maximum RF input power reaches up to 100 W for a continuous wave signal, and the switching time is about 12 μs), and the unblanking of the power amplifier. The RF signal generator (NI PXI-5422, National Instruments, Austin, TX, USA) produces a continuous wave signal. This RF signal is shaped by the SPDT 1 to form an RF pulse sequence and amplified by a power amplifier (TwinPulse400, Tomco, Stepney, Australia), then passes through the high power switch into the NMR probe. When each excitation pulse is over, the other channel of the high power switch is unblanked for signal reception. The generated NMR signal is amplified by a preamplifier (ZFL-1000LN +, Mini-circuits, New York, NY, USA), then digitized by a high-resolution digitizer (NI PXI-5122, National Instruments, Austin, TX, USA) with 100 MS/s and recorded by a disk array (HDD-8264 RAID, National Instruments, Austin, TX, USA). The SPDT 2 and 3 are used to protect the receiver during the pulse. All components are clocked by the 10 MHz reference clock of the PXI system.

In pulsed NMR experiments, the nutation angle of the macroscopic magnetic moment is mainly determined by the RF excitation field strength $B_{1}$ and the pulse width τ. When the nutation angle is π/2, the macroscopic magnetic moment is flipped to the *xy*-plane, meanwhile, the NMR signal strength reaches a maximum. Assuming an uniform magnetic field occupying the entire coil volume V, the width of a π/2 pulse is approximately given by [12] (pp. 38–39):

(2) $$\tau_{\pi /2}=\frac{\pi }{\gamma }\sqrt{\frac{\omega_{0}V}{2\mu_{0}QP}}$$

Under the following conditions: the resonance angular frequency $\omega_{0}=2\pi \times 24.652$ MHz, the volume of the coil $V=157\times 10^{-9}$ m^3^, the vacuum permeability $\mu_{0}=4\pi \times 10^{-7}$ H/m, the quality factor Q=35, and the RF power P=100 W, the π/2 RF pulse width calculated using Equation (2) is about 0.62 µs. Considering the inhomogeneity of RF excitation field, the calculated value is always smaller than the actual value. Therefore, this value can only provide a reference for the selection of π/2 pulse width. Experimental results showed that the pulse width of 1 µs is an appropriate choice.

**Figure 2:**
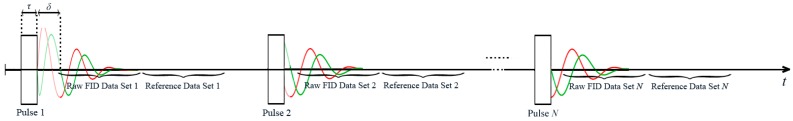
Sketch of the pulse sequence and the data selection.

The pulse sequence used here is a train of identical equidistant RF pulses derived from the RF carrier running at 24.65 MHz. The duration of RF pulse is 1 µs, and the pulse repetition time is 1 ms. NMR data with a temporal length of 30 ms is recorded, which contains 29 integral FID signals. The dead time δ during the interval from the end of the RF pulse to the start of the signal acquisition is 15 µs. In order to guarantee a high correlation of the interferences between raw FID data and reference data, the selected reference signal is an adjacent data set after the corresponding FID data set, as illustrated in Figure 2.

The NMR sample is a saturated CuSO_4_ aqueous solution (mass fraction about 20% at 300 K). The addition of paramagnetic salt can effectively accelerate the relaxation rates of ^1^H nuclei, thus avoiding the saturation of NMR sample in the process of pulse sequence excitation. The spin-lattice relaxation time $T_{1}$ and the spin-spin relaxation time $T_{2}$ of ^1^H are approximately 0.78 ms and 0.60 ms at 300 K, respectively [16]. Taking into account of the spatial inhomogeneity of the applied magnetic field, the apparent relaxation time $T_{2}^{*}$ [17] (pp. 50–52) will be shorter. The selected temporal length of the FID data set and the reference data set for signal processing are both 0.45 ms.

**Figure 3:**
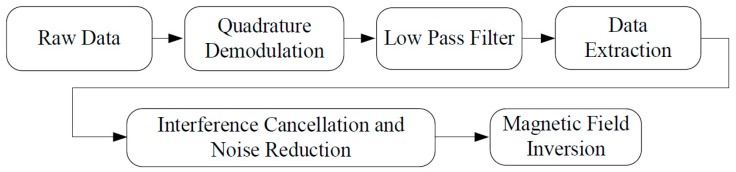
NMR data processing flow chart. The demodulation frequency is 24.65 MHz. The pass band of the digital low pass filter is 0–600 kHz, and the cutoff frequency is 1 MHz.

All data processing is done on PC using a self-written Matlab program. The data processing flow is shown in Figure 3. The FID data and reference data are firstly demodulated with quadrature reference signals, and then, these signals are filtered and extracted. After these pre-processing procedures, the number of sample points is dramatically reduced from 45,000 to 900, which provides a convenient for subsequent matrix operations.

**Figure 4:**
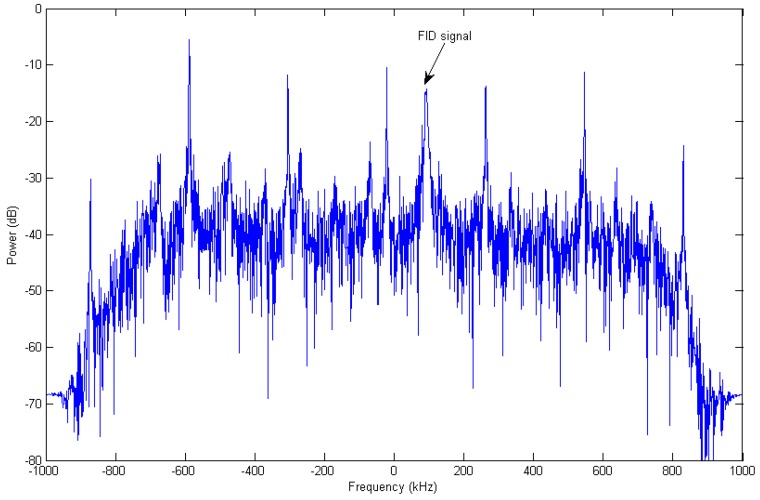
Spectrum of one FID data set of a low SIR. The data has been pre-processed (demodulation, low pass filtering and extraction). The FID signal peak is marked; others are RFI peaks. Obviously, the spectral width of the FID signal is broader than that of RFI.

Unfortunately, as shown in Figure 4, there are many large frequency-discrete comb interference peaks with a frequency interval nearly 284 kHz in the 4–38 MHz range. Moreover, this RFI is not a strictly stationary signal, as its frequency varies slowly over time. After testing, we found that this RFI mainly derives from the power supply component of our home-made high power SPDT switch. Because the time-dependent magnetic field strength is calculated from the spectral peak frequency of FID signal, a deviation will occur in final magnetic field fitting if the interference peak amplitude is comparable with that of NMR signal in certain low SINR data sets. In order to deal with this problem conveniently and effectively, a new interference cancellation algorithm based on singular value decomposition is proposed.

## 3. SVD-Based Interference Cancellation and Noise Reduction Method

Supposed the FID data set is $x=\left\{x_{1},x_{2},\cdots ,x_{l}\right\}$ and the reference data set is $y=\left\{y_{1},y_{2},\cdots ,y_{l}\right\}$. The vectors x and y can be used to construct Hankel matrices of dimension m×n as follows:

(3) $$A_{1}=\left[\begin{array}{cccc} x_{1} & x_{2} & \cdots & x_{n}\\ x_{2} & x_{3} & \cdots & x_{n+1}\\ \vdots & \vdots & \ddots & \vdots \\ x_{m} & x_{m+1} & \cdots & x_{l} \end{array}\right]$$

(4) $$A_{2}=\left[\begin{array}{cccc} y_{1} & y_{2} & \cdots & y_{n}\\ y_{2} & y_{3} & \cdots & y_{n+1}\\ \vdots & \vdots & \ddots & \vdots \\ y_{m} & y_{m+1} & \cdots & y_{l} \end{array}\right]$$

where, *m* is the number of rows of the Hankel matrix, which is usually taken half the total length of the data, *i.e.*, m=[l/2]. The floor function [l/2] gives the largest integer less than or equal to l/2. In addition, *m* and *n* subject to the constraint m+n−1=l. Applying singular value decomposition to matrices $A_{1}$ and $A_{2}$, and we get:

(5) $$A_{1}=U_{1}\Sigma_{1}{V_{1}}^{T}$$

(6) $$A_{2}=U_{2}\Sigma_{2}{V_{2}}^{T}$$

In Equations (5) and (6), the left eigenvector matrix U and the transposition of the right eigenvector matrix $V^{T}$ are the orthogonal matrices of dimension m×m and n×n, respectively; the main diagonal elements of the diagonal matrix Σ are called the singular values of A, which are non-negative and arranged in descending order.

The FID data set x and the reference data set y both contain RFI. However, the actual correlation between RFI in x and RFI in y is affected by a variety of hardware factors. Therfore, we plan to use the correlation between the columns of $U_{1}$ and $U_{2}$ to distinguish RFI from the FID signal. The linear correlation coefficient $\gamma_{ij}$ between the *i*-th column vector $u_{1i}$ of left singular matrix $U_{1}$ and the *j*-th column vector $u_{2j}$ of matrix $U_{2}$ can be defined by the Pearson correlation coefficient [18] (pp. 37–40):

(7) $$\gamma_{ij}=\frac{\mathrm{cov}(u_{1i},u_{2j})}{\sigma_{u_{1i}}\sigma_{u_{2j}}}$$

where the value range of subscript *i* and *j* is from 1 to *m*. $\mathrm{cov}(u_{1i},u_{2j})$ is the covariance of $u_{1i}$ and $u_{2j}$. $\sigma_{u_{1i}}$ and $\sigma_{u_{2j}}$ are the standard deviation of $u_{1i}$ and $u_{2j}$, respectively. Thus, we can obtain a correlation matrix *C*:

(8) $$C=\left[\begin{array}{cccc} \left|\gamma_{11}\right| & \left|\gamma_{12}\right| & \cdots & \left|\gamma_{1m}\right|\\ \left|\gamma_{21}\right| & \left|\gamma_{22}\right| & \cdots & \left|\gamma_{2m}\right|\\ \vdots & \vdots & \ddots & \vdots \\ \left|\gamma_{m1}\right| & \gamma_{m2} & \cdots & \left|\gamma_{mm}\right| \end{array}\right]$$

Assuming the FID signal mainly presents in the data set x. If the maximum value in the *k*-th row of the correlation matrix C is greater than a given threshold value $k_{in}$, it means the *k*-th column vector of matrix $U_{1}$ belongs to the interference subspace; the corresponding singular value $\lambda_{k}$ of the diagonal matrix $\Sigma_{1}$ can be set to zero. Thus, the original diagonal matrix $\Sigma_{1}$ is replaced by a new matrix $\Sigma_{1}^{\prime }$.

In the diagonal matrix $\Sigma_{1}^{\prime }$, several previous large singular values corresponding to RFI has been removed, while the smaller singular values, mainly represent the noise component, still exist. The SINR of NMR data can be further improved by removing the small singular values. Assuming the FID signal is a single frequency signal, the random noise can be reduced with the greatest degree by preserving only the largest one of the remaining singular values in $\Sigma_{1}^{\prime }$, and we can obtain a new diagonal matrix $\Sigma_{1}^{\prime \prime }$.

After the ICNR processing, the estimated matrix $A_{1}^{\prime }$ of $A_{1}$ can be reconstituted according to the following formula:

(9) $$A^{\prime }=U_{1}\Sigma_{1}^{\prime \prime }{V_{1}}^{T}$$

Generally, the reconstruction matrix $A_{1}^{\prime }$ is no longer a Hankel matrix. The estimated signal $x^{\prime }=\left\{x_{1}^{\prime },x_{2}^{\prime },\cdots ,x_{l}^{\prime }\right\}$ can be obtained by averaging the diagonal elements of the reconstruction matrix:

(10) $$x_{i}^{\prime }=\frac{1}{s-p+1}\sum_{j=1}^{s}{Α}_{i-j+1,j}^{\prime }$$

where, s=min(n,i) and p=max(1,i−m+1).

**Figure 5:**
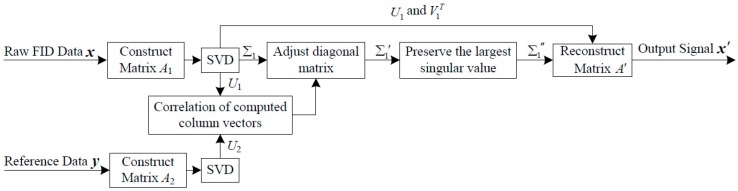
Schematic overview of the ICNR algorithm based on singular value decomposition.

In summary, the architecture of the proposed SVD-based ICNR algorithm in this article is shown in Figure 5.

## 4. Results and Discussion

**Figure 6:**
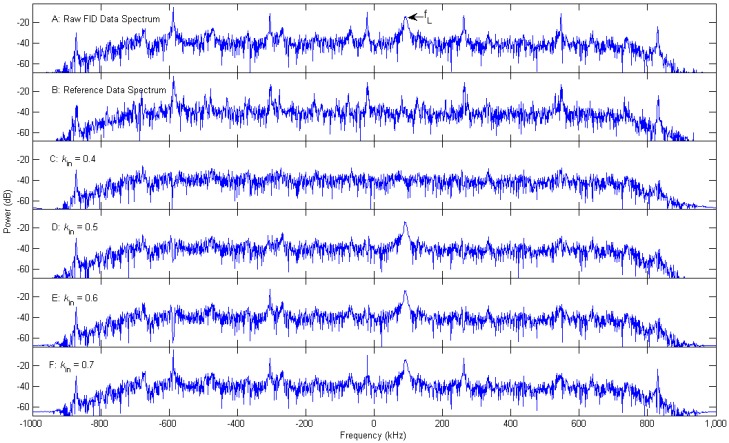
Fourier amplitude spectra of (**A**) the FID data; (**B**) the corresponding reference data; and various spectra after interference cancellation with different threshold value (**C**) $k_{in}$ = 0.4; (**D**) $k_{in}$ = 0.5; (**E**) $k_{in}$ = 0.6; (**F**) $k_{in}$ = 0.7, respectively.

A plot of the interference cancellation effect of different threshold values is shown in Figure 6. The upper two parts of Figure 6 are the Fourier amplitude spectra of one of the 29 FID data sets and the corresponding reference data set, respectively. It shows that the RFI in the two data sets are correlated. The lower four parts show various spectra after interference cancellation with a threshold value $k_{in}$ = 0.4, 0.5, 0.6, 0.7, respectively. This figure clearly demonstrates that, the FID signal is cancelled simultaneously when the selected threshold value is too low, e.g., $k_{in}$ = 0.4. It is unable to suppress all of the interference peaks with a too high threshold value, such as $k_{in}$ = 0.6 or 0.7, which implies that the actual correlation between RFI in FID data set and that in reference data set is smaller than these threshold value. When the threshold value $k_{in}$ = 0.5, the interference can be effectively cancelled and the FID signal is better preserved. It should be noted that, the first RFI peak on the left is not suppressed even when $k_{in}$ = 0.4, probably because the power of this interference component after filtering is comparable with that of the noise in the pass band.

This means that, to achieve a good result using the above SVD-based ICNR algorithm, the key factor is to choose an appropriate threshold value $k_{in}$. The optimal threshold value should be bigger than the correlation between FID signal in the FID data set and RFI near the Larmor frequency in reference data set, meanwhile, smaller than the correlation between the RFI components need to be cancelled in these two data sets. However, we have not found a suitable way to quantitatively assess their correlations. At the present stage, the selection of the threshold value is primarily determined by the interference cancellation effect.

**Figure 7:**
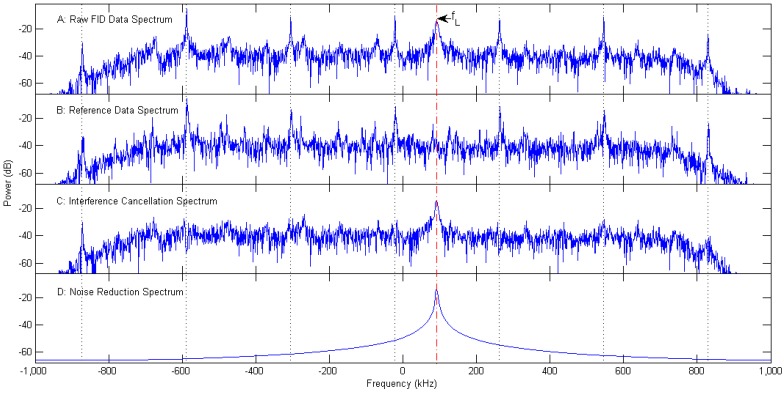
The effect of interference cancellation and noise suppression. The spectra of (**A**) the raw FID data; (**B**) the reference data; (**C**) the FID data after interference cancellation with a threshold value of 0.5; (**D**) noise suppression by preserving only the largest one of the remaining singular values.

In Figure 7, the spectra of the FID data, the reference data, and the corresponding spectra of the FID data after interference cancellation and noise suppression are shown. Figure 7C shows that, an interference cancellation effect of approximately 20–30 dB can be reached across the interesting frequency band using our SVD-based interference cancellation algorithm, meanwhile, the FID signal peak at the Larmor frequency is left undisturbed after interference cancellation. Thus, the spectral peak amplitude of FID signal is significantly higher than those of RFI. It effectively avoids a wrong selection of the spectral peak frequency of FID signal in the subsequent data processing procedure. After interference cancellation, the FID signal is still corrupted by random noise. To further improve the SINR, some of the small singular values are removed to reduce the noise. The spectrum of preserving only the largest one of the remaining singular values after interference cancellation is given in Figure 7D.

**Figure 8:**
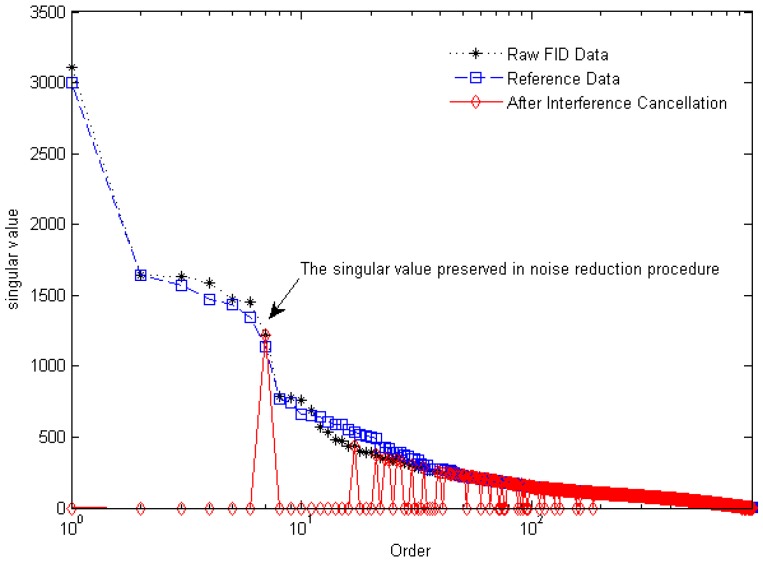
Singular value plots of Figure 7A–C. The marked singular value corresponds to Figure 7D.

**Figure 9:**
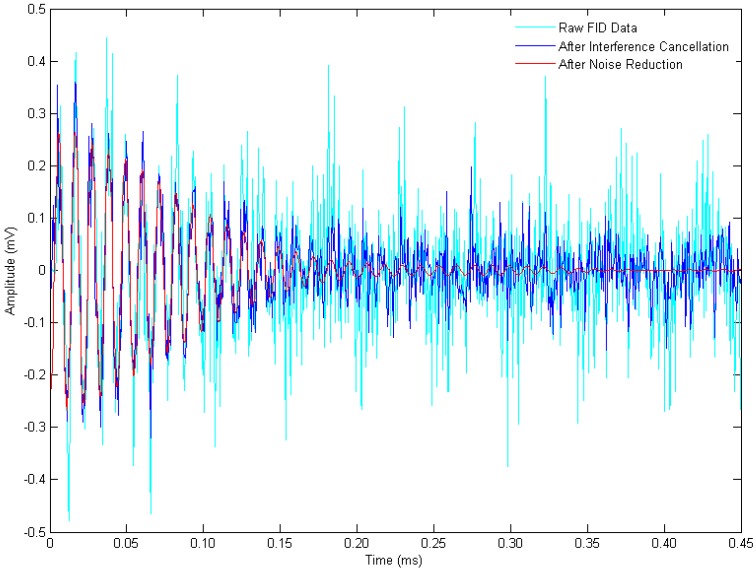
The waveforms of raw FID data (cyan), and the data after interference cancellation (blue) and noise suppression (red).

Figure 8 gives the singular value curves of the FID data, the reference data, and the data after interference cancellation. All of the first fifteen largest singular values, except the 7th singular value (marked in Figure 8), are identified and set to zero by the interference cancellation algorithm. And then, only this marked singular value is preserved to reconstitute the FID signal. The corresponding spectrum, whose peak frequency is consistent with the Larmor frequency, is shown in Figure 7D, and the waveform is illustrated in Figure 9 (red curve).

Time-series plots of Figure 7 are shown in Figure 9. In this figure, it is obviously easier to observe a FID signal after data processing, preliminarily verifying the validity of our SVD-based ICNR algorithm. The rapid decay of FID signal illustrates that the apparent relaxation time $T_{2}^{*}$ is shorter than 0.15 ms, indicating a rather inhomogeneous applied magnetic field.

**Figure 10:**
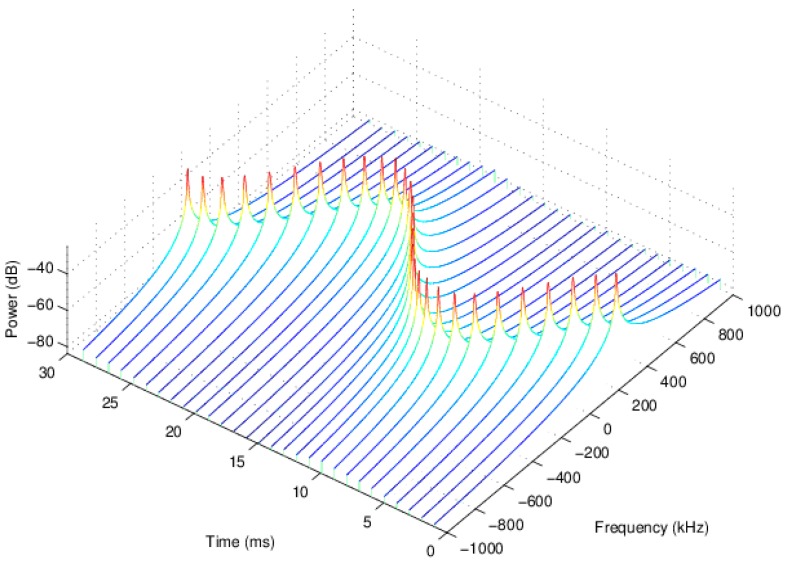
The waterfall graph of all 29 NMR spectra.

The NMR spectra of all 29 FID signals after ICNR are shown in Figure 10. It can be seen clearly that the spectral peak frequency of each FID signal varies sinusoidally, confirming the feasibility of the magnetic field measurement scheme.

**Figure 11:**
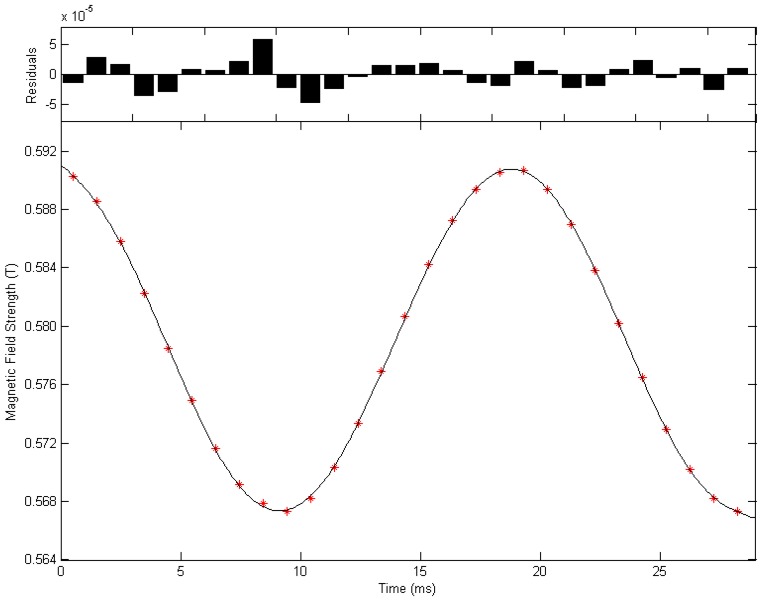
Fitting curve of the alternating magnetic field when the operating voltage is 250 V and residual error.

The magnetic field strength at each pulse excitation moment can be calculated from the spectral peak frequency of the corresponding FID signal set. All of the parameters in Equation (1) can be estimated by fitting the 29 data points of magnetic field strength using a least square method. Figure 11 shows the fitting curve of the alternating magnetic field and the corresponding residual error. Fitting result indicates that, the permanent magnetic field $B_{z0}$ is 0.5798 T; the amplitude of the alternating magnetic field *B_zp_* is 0.0117 T when the operating voltage is 250 V; angular frequency $\omega_{z}$ is 2π×50.0676 rad/s, which is close to the nominal alternating current frequency 50 Hz; the initial phase $\varphi_{z0}$ is 0.0066 rad.

## 5. Conclusions

In this paper, the NMR experiment in time-dependent magnetic fields, including the experimental setup and the magnetic field measurement scheme, was introduced. The NMR method can achieve higher measurement accuracy, larger measurement range, as well as has a faster response speed than other techniques used in measurement of time-dependent magnetic fields, such as flux meter, magneto-resistance, magneto-optical, Hall effect, *etc.* [19]. In addition, to improve the SINR, a new SVD-based ICNR algorithm was proposed, and used in processing measured NMR data. The raw FID data was corrupted by additive RFI and noise; the selected reference data, which is close to the FID data, comprised only correlated interference and uncorrelated noise. Applying SVD to the constructed Hankel matrices of these two signals, and then removing the singular values corresponding to the interference and noise components using our SVD-based ICNR algorithm, the SINR of raw NMR data was significantly improved. The interference cancellation result showed that the amplitude of interference peaks can be suppressed around 20 to 30 dB. Based on our investigation, we found that the choice of the optimal threshold value for interference cancellation depends on the correlation of the interference components between the two data sets.

Compared with other interference cancellation methods, the proposed SVD-based interference cancellation algorithm in this article has a wider range of applications, such as for cancelling non-stationary interference, wideband interference, or interference with a comparable or even smaller power, and does not require an iterative process and is suitable for real-time processing. However, there are also some limitations of our algorithm.

The main problem is that there is no mature and effective way to determine the optimal threshold value. The current version of our SVD-based ICNR technique needs a user-defined threshold for retaining or eliminating the singular values. This is an important limitation of the technique at this stage of development. In this article, the selection of the optimal threshold value mainly relies on the actual cancelling effect.

In addition, in the period of rapid change of an applied time-dependent magnetic field, the FID signal frequency is dramatically modulated by the magnetic field, resulting in spectral line broadening [20]. Preserving only the first largest singular value in the noise reduction procedure can naturally achieve a highest improvement in SINR. However, the reconstituted waveform of FID signal using this strategy may produce distortion if the NMR signal has a broad spectrum, although such a distortion does not change the spectral peak frequency and have no effect on the final magnetic field fitting result. To avoid distortion of the FID waveform in some special application environments, e.g., a pulsed magnetic field of a high field strength and a short duration, some other criteria of determining the optimal de-noising order can be used as a reference, such as the increment of the singular entropy [21], the difference spectrum of the singular values [22], the curvature spectrum of the increment of singular entropy [23], *etc*.

Finally, in order to get a high correlated reference signal, besides sampling the raw FID data, we also need to record a reference data set of the same length during two excitation pulses. This strategy makes the available number of FID signal sets is reduced by half in the same data acquisition time, which may result in an accuracy reduction of data fitting.

At the end, we fitted the 29 processed experimental data points using a least square method and estimated all parameters of the alternating magnetic field. The results are consistent with the measured values using a gauss meter, indicating the validity of the ICNR algorithm and magnetic field measurement scheme.
